# Gastric Adenocarcinoma: A Multimodal Approach

**DOI:** 10.3389/fsurg.2017.00042

**Published:** 2017-08-03

**Authors:** Humair S. Quadri, Brandon G. Smaglo, Shannon J. Morales, Anna Chloe Phillips, Aimee D. Martin, Walid M. Chalhoub, Nadim G. Haddad, Keith R. Unger, Angela D. Levy, Waddah B. Al-Refaie

**Affiliations:** ^1^Department of Surgery, MedStar Georgetown University Hospital, Georgetown Lombardi Comprehensive Cancer Center, Washington, DC, United States

**Keywords:** gastric cancer, oncology, multimodal therapy, gastric adenocarcinoma, multidisciplinary approach, gastrectomy, radiotherapy, chemotherapy

## Abstract

Despite its declining incidence, gastric cancer (GC) remains a leading cause of cancer-related deaths worldwide. A multimodal approach to GC is critical to ensure optimal patient outcomes. Pretherapy fine resolution contrast-enhanced cross-sectional imaging, endoscopic ultrasound and staging laparoscopy play an important role in patients with newly diagnosed ostensibly operable GC to avoid unnecessary non-therapeutic laparotomies. Currently, margin negative gastrectomy and adequate lymphadenectomy performed at high volume hospitals remain the backbone of GC treatment. Importantly, adequate GC surgery should be integrated in the setting of a multimodal treatment approach. Treatment for advanced GC continues to expand with the emergence of additional lines of systemic and targeted therapies.

## Introduction

Gastric cancer (GC) is the third leading cause of cancer-related deaths worldwide and wide variations exist throughout the world (1). For example, Japan and Eastern Asia have a higher incidence (approximately 18–25 cases/100,000) than Europe and North America (approximately 8–10 cases/100,000) (2). In contrast, the incidence of GC in the US is steadily declining, representing the 15th most prevalent cancer. It was estimated that 24,500 patients would be diagnosed with GC in 2015, and of those, men were twice as likely to develop GC as women. With an estimated 5-year survival of 29%, over 10,000 Americans are forecast to die in 2016 from GC (3).

## Presenting Features

Gastric cancer can present with a variety of non-specific gastrointestinal (GI) symptoms that can easily mimic benign conditions such as gastroesophageal reflux or peptic ulcer disease. This makes the diagnosis of GC easily overlooked, especially when it is relatively uncommon in the US. Patients who may have more defined symptoms of early satiety, dysphagia, or weight loss should undergo further evaluation with an upper endoscopy (4). Those that progress to more advanced stages may present with a palpable abdominal mass, cachexia, ascites, or bowel obstruction. The findings of a gastric primary tumor that has metastasized to the ovary are known as a Krukenberg tumor (5). The classic physical examination findings of metastatic disease, such as an enlarged supraclavicular node (Virchow’s node), periumbilical lymph node (Sister Mary-Joseph’s node), or drop metastasis in the pouch of Douglas (Blumer’s shelf), are rarely found on physical examination (5). Unfortunately, most physical examination findings are usually non-specific and cannot be relied upon for a definitive diagnosis.

## GC Risk Factors

Several risk factors are associated with GC. One of the most noted risk factors is infection with *Helicobacter pylori*, which is predominant in patients in developing countries and is more often seen with distal (noncardia) gastric tumors. The prevalence of *H. pylori* in the US is less than 20% at 20 years old and approximately 50% at 50 years. Studies have reported that there is a high incidence of *H. pylori* infection in patients with GC with epidemiological studies reporting an estimated 75% attributable risk of developing GC with long standing *H. pylori* infection (6–8). The Epstein-Barr virus (EBV) has also been associated with GC. The integration of EBV and its viral genes have been shown to be a cause of carcinogenesis (9). Diet also serves as a risk factor for developing GC. Specifically, diets that include increased salt, smoked, or poorly preserved foods, nitrates, nitrites, and secondary amines have been known to play a role in increasing risk. The World Health Organization (WHO) released a statement about the increased risk of GC with a large intake of salt preserved foods (10). Conversely, diets high in raw vegetables, fresh fruits, vitamin C, vitamin A, calcium, and antioxidants are associated with a decreased risk of the development of GC (4). Cigarette smoking is another major environmental factor that is associated with a two- to threefold increase in GC, and heavy alcohol consumption may also pose an increased risk (5). Some suggest that ethnicity may play a role as a risk factor. While certain ethnic groups may be associated with a higher incidence of GC, environmental factors are more strongly considered (11).

## Hereditary GC Syndromes

Approximately 10% of GC s cluster in families and 1 to 3% are due to an inherited cancer syndrome (12–14). Features suggestive of hereditary risk include GC in two or more first-degree relatives and/or second-degree relatives, GC or related cancers in multiple generations, signet ring cell histology, and early age of onset (<45 years) (15). Careful attention to the pathology of GC in the individual is critical in assessing risk. Hereditary diffuse gastric cancer (HDGC) is the most recognized form of hereditary GC, while intestinal type GC can be seen in several different hereditary cancer syndromes, including Lynch syndrome (LS), Li-Fraumeni syndrome (LFS), familial adenomatous polyposis (FAP), Peutz-Jeghers syndrome (PJS), juvenile polyposis syndrome (JPS), *MUTYH-*associated polyposis (MAP), and hereditary breast and ovarian cancer syndrome (HBOC) (16). Therefore, careful attention to family history is needed to ensure the most accurate risk assessment and genetic testing approach. A cancer genetics professional, primarily a genetic counselor, can help obtain this history, formulate a differential, and coordinate laboratory testing. Additionally, these professionals are trained to observe physical features, including cutaneous findings that can be associated with some of the above syndromes. They can also discuss options for managing risk. Below, we summarize the main syndromes related to hereditary GC risk.

## Hereditary Diffuse Gastric Cancer

Hereditary diffuse gastric cancer was one of the first hereditary GC syndrome to be identified (17). It is caused by germline mutations in *CDH1*, the gene that encodes the E-cadherin protein (17). Individuals with HDGC have an increased risk of developing diffuse gastric cancer (DGC), 70% for men (95% CI 59–80%) and 56% for women (95% CI 44–69%); lobular breast cancer (LBC) 42% (95% CI 23–68%) by the age of 80 years; and possibly colon cancer (18). The average age of onset for DGC is 38 years (range 14–69 years), and LBC can occur premenopausally (17, 19). Individuals at risk for HDGC meet at least one of the criteria outlined in Table 1, established by the International Gastric Cancer Linkage Consortium (IGCLC) in 2010 and updated by van der Post et al. (20, 21). Germline *CDH1* testing is recommended in these cases. *CDH1* detection rates in areas with a low incidence of GC, such as North American and Western Europe, are estimated at 10–18% (20). This is much lower than previous reported estimates. Therefore, the vast majority of individuals suspicious for HDGC will test negative for mutations in *CDH1*.

**Table 1:** International Gastric Cancer Linkage Consortium (IGCLC) criteria for HDGC.

Established criteria^a^	Families in whom testing could be considered^a^
Families with two or more patients with gastric cancer at any age, one confirmed DGC	Bilateral LBC or family history of 2 or more cases of LBC < 50
Individuals with DGC before the age of 40	A personal or family history of cleft lip/palate in a person with DGC
Families with diagnoses of both DGC and LBC (one diagnosis before the age of 50)	*In situ* signet ring cells and/or pagetoid spread of signet ring cells

*^a^Including first- and second-degree relatives*.

*HDGC, hereditary diffuse gastric cancer; DGC, diffuse gastric cancer; LBC, lobular breast cancer*.

Due to the relative ineffectiveness of endoscopic surveillance for detection of DGC, and the early age of onset, total gastrectomy (TG) for GC patients or prophylactic gastrectomy (for at-risk family members) is recommended for *CDH1* carriers (20). Guidelines are less clear for managing families who meet established criteria for which no mutation is present. In this case, families should be referred to an expert center for discussion of endoscopic surveillance (20). Mutation-positive females with DGC (and those at-risk) are also encouraged to obtain high-risk breast cancer surveillance through a specialized center. Although signet ring colon cancer has also been reported in HDGC families, the risk has not been well established (22). However, screening for colon cancer may be initiated by age 40 in families with colon cancer (21).

## Other Syndromes

A higher incidence of GC has been noted in several other hereditary cancer syndromes including LS, LFS, FAP, PJS, JPS, MAP, and HBOC. Because GC is typically not the defining feature of these syndromes, careful consideration should be paid to polyp history, age of cancer onset, tumor histology, as well as family history. GC in LS occurs at a frequency of 1.6% and is typically of the intestinal type (23). However, in a small subset of individuals diffuse or mixed histology may be observed (23). Mutation carriers can have up to a 13% risk of developing GC, depending on the mismatch repair (MMR) gene mutation present in the family (24, 25). LS is defined by higher rates of colon cancer (10–70%) and gynecologic malignancy (up to 60% of uterine cancer and 12% of ovarian cancer) in female carriers (26). LFS is typically defined by early onset sarcoma, breast cancer, brain tumor, and adrenal cortical carcinoma. GC has been observed at a frequency of 4.9% and is mainly of the intestinal type, although diffuse type has been reported (27). The lifetime risk for GC in LFS is <3% with a mean age of diagnosis at 36 years (range 24–74 years), with most patients presenting prior to age 50 (27, 28). FAP is caused by mutations in the *APC* gene and leads to severe polyposis in mutation carriers as well as a 70–100% risk of colon cancer, depending on the location of the mutation and presentation in the family. Individuals with FAP also have an increased risk of developing adenomas in the upper GI tract, especially in the duodenum, and if untreated, this can progress to malignant disease in around 5% of cases (29). Fundic polyps are the most prevalent gastric polyp in FAP, and focal low-grade dysplasia is commonly seen; however, malignant transformation of gastric adenomas in individuals with FAP is rare (30, 31).

Gastric cancer has also been linked to other rare polyposis syndromes such as MAP, PJS, and JP; however, these syndromes are mainly defined by colonic and upper intestinal polyposis, colon cancer, as well as extra-intestinal cancers. Table 2 details these features with highest risk of GC seen in PJS. In regards to HBOC, early studies showed a promising association between GC and *BRCA1* and *BRCA2* mutations, however, the association has appeared to diminish in more recent studies (32–34). Nevertheless, in early onset breast/GC families that are negative for mutations in *TP53*, MMR genes, and *CDH1*, it may not be unreasonable to examine *BRCA1* and *BRCA2*.

**Table 2:** Hereditary cancer syndromes.

Hereditary cancer syndrome	Responsible gene(s)	Inheritance	Lifetime risk of gastric cancer	Other malignancies
Lynch syndrome (LS)	*MLH1, MSH2, MSH6, PMS2, EPCAM*	AD	1–13%	Uterus, ovary, hepatobiliary, urological, pancreas, brain, small bowel (3–25)
Li-Fraumeni syndrome (LFS)	*TP53*	AD	<3%	Sarcoma, breast, brain, adrenal cortical (2, 6)
Peutz-Jeghers syndrome (PJS)	*STK11*	AD	29%	Breast, pancreas, small bowel, lung, ovary, testis, cervix (27)
Familial adenomatous polyposis (FAP)	*APC*	AD	~0.6%	Thyroid, duodenum, small bowel, brain (28)
Juvenile polyposis syndrome (JPS)	*SMAD4, BMPR1A*	AD	30% in those with *SMAD4* mutations	Small bowel, pancreas (2, 9, 30)
*MUTYH-*associated adenomatous polyposis (MAP)	*MUTYH*	AR	No statistically significant data available	Thyroid, duodenum (1, 6)
Hereditary breast and ovarian cancer syndrome (HBOC)	*BRCA1, BRCA2*	AD	Undefined	Breast, ovary, pancreas, melanoma (31–33)

*AD, autosomal dominant; AR, autosomal recessive*.

### Diagnosis

#### History and Physical Examination

A patient who is suspected to have symptoms of GC should be evaluated with a thorough history and physical examination. Notable questions of interest in the history should include unintentional weight loss, anorexia, early satiety, vomiting, bleeding, epigastric burning, pain, or discomfort. Social factors such as tobacco and alcohol use and the consumption of large amounts of nitrate rich or smoked/preserved foods should also be taken into consideration for possible risk factors. A previous history of *H. pylori* infection and a family history of GC are also important (4). Furthermore, determining a patient’s performance status is also critical to predict their tolerance to various oncologic therapies.

#### Laboratory Workup

The diagnosis and pretherapy evaluation of patients with GC should include:

- Complete blood count (CBC): to evaluate and treat GC - or treatment-related anemia.
- Basic metabolic panel (BMP): to detect electrolyte abnormalities, especially in gastric outlet obstruction, and to detect renal functional abnormalities prior to receiving contrast-enhanced imaging and systemic therapy.
- Liver function panel: check prior to induction of preoperative systemic therapy.
- Albumin and prealbumin: to detect malnutrition, since approximately 30–80% of patients diagnosed with GC are malnourished (34).

### Genetic Testing for Hereditary GC

Although genetic testing may not be necessary for the diagnosis of some hereditary GI syndromes (i.e., FAP can be diagnosed through a clinical diagnosis of polyposis), it can be beneficial for clinicians and their patients in gaging the severity of the disease (attenuated FAP vs. classic FAP) and the risk for extra-intestinal cancer, and to help identify mutation carriers in the family. Genetic testing is available for each syndrome listed in Table 2 through several commercial laboratories in the US and worldwide. Individuals presenting with DGC should be referred for *CDH1* testing and if the results are negative, additional testing options may be considered. Genetic testing for individuals with intestinal type GC may be dependent on other factors in their personal and family history.

The traditional approach to genetic testing typically involves analyzing a single gene or a few related genes, such as in the case of LS, based on cancers observed in families. However, because many of these syndromes have overlapping features and because a good percentage of people (30–50%) presenting for genetic testing will not have a family history significant enough to warrant testing, this approach may not identify all mutation carriers (35). In patients with GC who do not have a family history of cancer or who have a family history that is limited, and for whom additional diagnostic procedures (colonoscopy) have been uninformative, a broader approach through a multigene panel may be beneficial and more economical. Patients should be counseled on these factors prior to testing, especially regarding the limitations of the technology, the possible implications of the test results for their personal health and their family members, the possible out-of-pocket costs, and the insurance implications.

## Diagnostic and Staging Modalities

### Upper GI Endoscopy

#### Screening

Since early detection of GCs can significantly improve outcomes, screening esophagogastroduodenoscopy (EGD) is performed in countries with a high prevalence of GC, such as Japan and South Korea. In regions outside of Asia with lower prevalence, endoscopic screening has not been shown to be beneficial for primary prevention in the general population (36). In patients with hereditary GI cancer syndromes, screening should be considered in certain cases (30). Those with adenomatous polyposis syndromes, such as FAP or MAP, screening EGD is recommended starting at age 25–30 years and repeated screening every 0.5–4 years according to the Spigelman stage of duodenal polyposis (37). Patients with LS should typically undergo initiation of EGD with biopsy at age 30–35, with treatment of any *H. pylori* infection and surveillance every 3–5 years. Evidence for screening EGD in hamartomatous polyposis syndromes, including Peutz-Jeghers syndrome, juvenile polyposis syndrome, Cowden syndrome, and serrated polyposis syndrome, is limited and should be considered on a case-by-case basis. As previously described in patients with HDGC, prophylactic TG is typically recommended at a young age, obviating the need for screening EGD.

#### Surveillance of Precancerous Lesions

When identified on EGD, gastric adenomas are removed *via* endoscopic resection (ER) in a similar fashion to adenomas found in the colon. All specimens should be sent for histologic analysis. The incidence of GC following resection of gastric adenomas is not clearly defined, but recent reports indicate that it is in the neighborhood of 3% (38). Given this uncertainty regarding the natural history of these lesions, optimal surveillance recommendations are similarly uncertain. Current recommendations are for repeat EGD in 1 year for adenomatous and hyperplastic lesions (39).

In addition to gastric adenomas, atrophic gastritis, intestinal metaplasia, and epithelial dysplasia confer an increased risk for the development of GC. When gastric histology reveals extensive atrophy or intestinal metaplasia, it is recommended to perform surveillance EGDs every 3 years with multiple non-targeted, or “mapping,” biopsies to assess for the development of dysplasia. In cases of mild or moderate atrophy or intestinal metaplasia, there is no clear role for endoscopic surveillance. In cases of low-grade dysplasia without an endoscopically identified lesion, patients should undergo repeat EGD at 1 year. When high-grade dysplasia is present, patients should undergo immediate repeat EGD evaluation, followed by EGD surveillance at 6-month to 1-year intervals (40).

In all of the above cases, there is a strong recommendation to eradicate *H. pylori* when it is present. Fundic gland polyps and inflammatory fibroid polyps typically do not require surveillance.

#### Endoscopic Appearance

**Figure 1:**
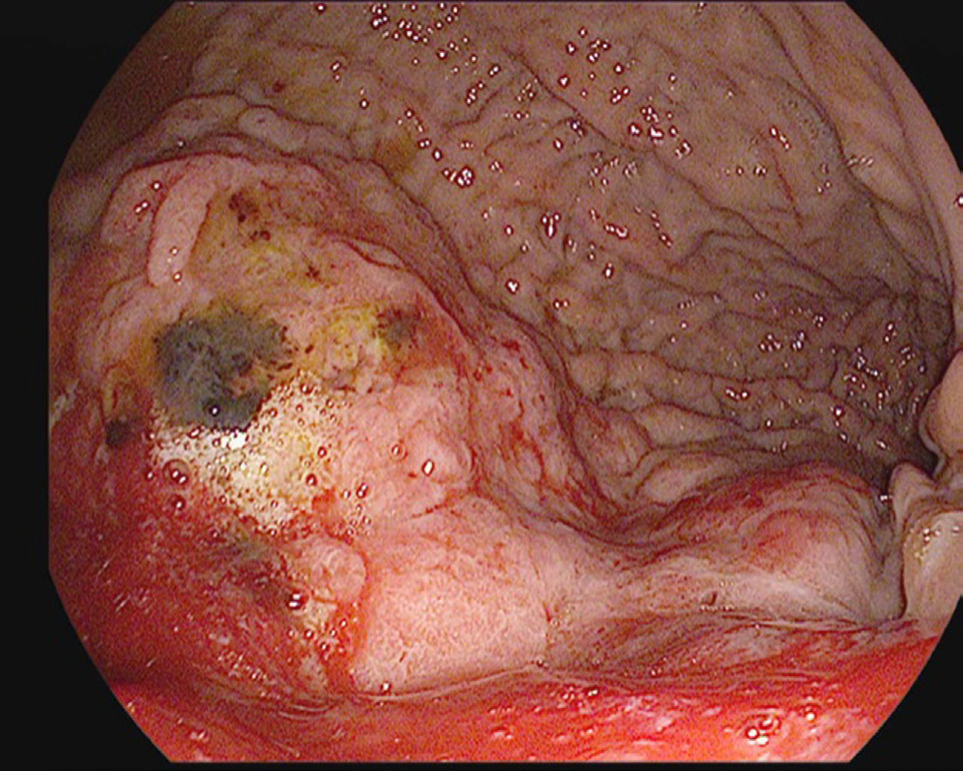
A large, friable, actively oozing fundic mass with extension into the cardia, found in a 69-year-old gentleman who underwent esophagogastroduodenoscopy (EGD) for anemia and melena. Pathology was consistent with gastric adenocarcinoma.

Screening or surveillance EGDs are performed in a clear field with a standardized procedure for mapping the stomach. Documentation is highly recommended to assure visualization of the entire mucosa of the stomach to avoid missing lesions. Detecting subtle changes in the gastric mucosa is often challenging. Features of a dysplastic or cancerous lesion include disruptions in the contour of the gastric mucosa; lesions, which can be pedunculated or sessile; and alterations in their vascular patterns (Figure 1). The Paris classification is used to further categorize the appearance of these lesions (Table 3) (41). Several techniques have been developed to assist with identifying such lesions, including magnifying endoscopy, chromoendoscopy, narrow band imaging, flexible spectral color enhancement endoscopy, and confocal laser endomicroscopy. These techniques have shown great promise in improving endoscopic detection of worrisome lesions and in guiding biopsy site selection (42).

**Table 3:** Paris classification.

Type	
0	Superficial polypoid, flat/depressed, or excavated tumors
1	Polypoid carcinomas, usually attached on a wide base
2	Ulcerated carcinomas with sharply demarcated and raised margins
3	Ulcerated, infiltrating carcinomas without definite limits
4	Non-ulcerated, diffusely infiltrating carcinomas
5	Unclassifiable advanced carcinomas

#### Diagnosis

In patients with a high suspicion for GC, EGD is the optimal means of diagnostic confirmation. This modality allows for direct visualization of the lesion as well as confirmation of the diagnosis *via* biopsy. Biopsy can be obtained in the form of tissue sent for histopathological assessment. Specimens are assessed according to WHO criteria or Lauren classification (43, 44).

The Lauren classification has defined two distinct histological types for gastric adenocarcinoma: intestinal and diffuse. An indeterminate type is also used to describe uncommon variants. Each type has been classified based on differences of cell morphology and pattern of growth. The intestinal type is characterized by glandular features and is usually well to moderately differentiated. On the other hand, the diffuse type is characterized by poorly differentiated cells that have signet ring features (44).

The intestinal type is usually associated with chronic gastritis and is associated more with geographic regions that have an increased risk of GC. This type of cancer is usually seen in older males with chronic inflammation secondary to *H. pylori* infections and external risk factors (smoking, alcohol, nitrates). The diffuse type is seen in younger patients and can be more aggressive, especially in cases where there is a genetic component (5).

The WHO in 2010 has also classified GC using its own system of four distinct histologic types: tubular, mucinous, papillary, and signet cell. Each type is also described based on the cell morphology and was created based on the current described patterns of carcinoma (43). Immunoprofiling is an emerging technique used to identify expression levels of certain proteins (MUC2, CDX2) as adverse prognostic indicators based on distinct phenotypes of GC. While immunoprofiling does have great promise, it is still in the early stages of prognostic evaluation using retrospective survival data from GC tissue samples (45).

### Endoscopic Ultrasound (EUS)

**Figure 2:**
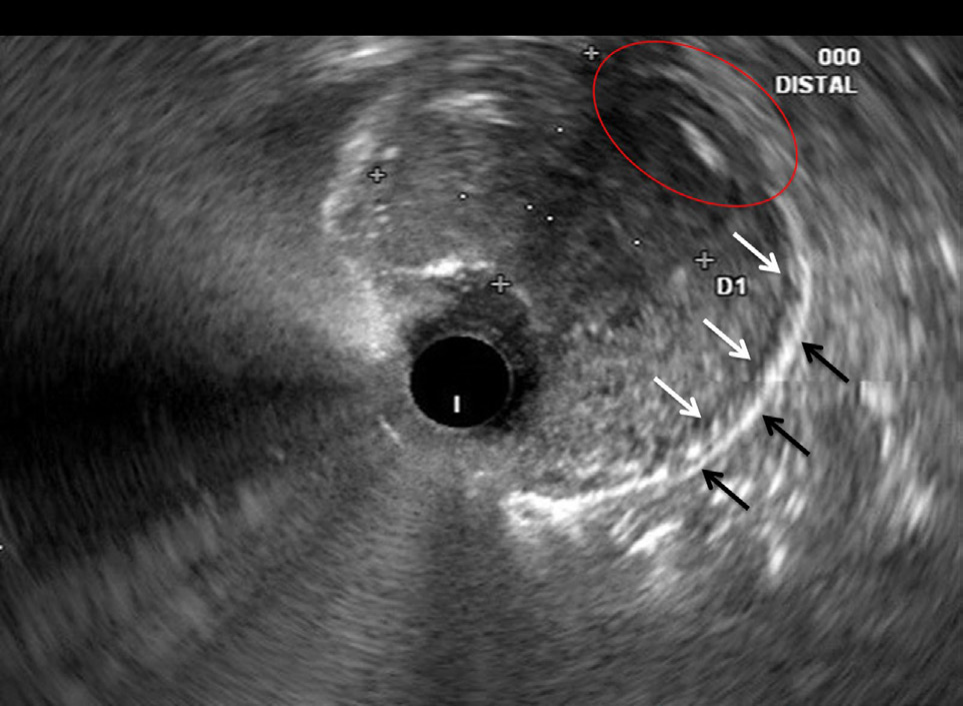
Correlative endoscopic ultrasound image revealing the muscularis propria (white arrows), serosa (black arrows), and an area in which the mass invades these layers (red oval). The mucosal and submucosal layers are obliterated by the mass and difficult to identify in this image.

In recent years, EUS has also emerged as an accurate staging tool in GC (46, 47). Assessment of T-stage consists of placing the EUS probe directly over the primary tumor in order to visualize its extent of invasion into the gastric wall (Figure 2). According to a Cochrane review, EUS can distinguish T1 (invasion of lamina propria, muscularis mucosae, or submucosa) from T2 (invasion of muscularis propria) lesions with a sensitivity of 85% and a specificity of 90%. Further, T1 and T2 (superficial) lesions can be differentiated from T3 and T4 (advanced) lesions with a sensitivity of 86% and a specificity of 90% (48).

To perform N-staging, EUS is used to search for nodal involvement in the paracardial, superior gastric, inferior gastric, and pancreaticoleinial regions. Lymph nodes are typically assessed according to their size and appearance, with hypoechoic, round, and well-demarcated lymph nodes typically considered to be positive. The sensitivity and specificity for identifying nodal involvement *via* EUS is 83 and 67%, respectively (48). While N-staging with EUS is less reliable than T-staging with EUS, it remains an informative modality that can impact treatment plans for patients with gastric adenocarcinoma (47, 48).

In the hands of an experienced endoscopist, EUS is beneficial in the locoregional staging of gastric adenocarcinoma in informing use of perioperative versus adjuvant therapy. Studies correlating EUS staging with postoperative staging indicated that EUS assessment of T- and N-stages was generally accurate and useful in guiding therapy despite some heterogeneity of the data (48).

### Contrast-Enhanced Cross-sectional Imaging

**Figure 3:**
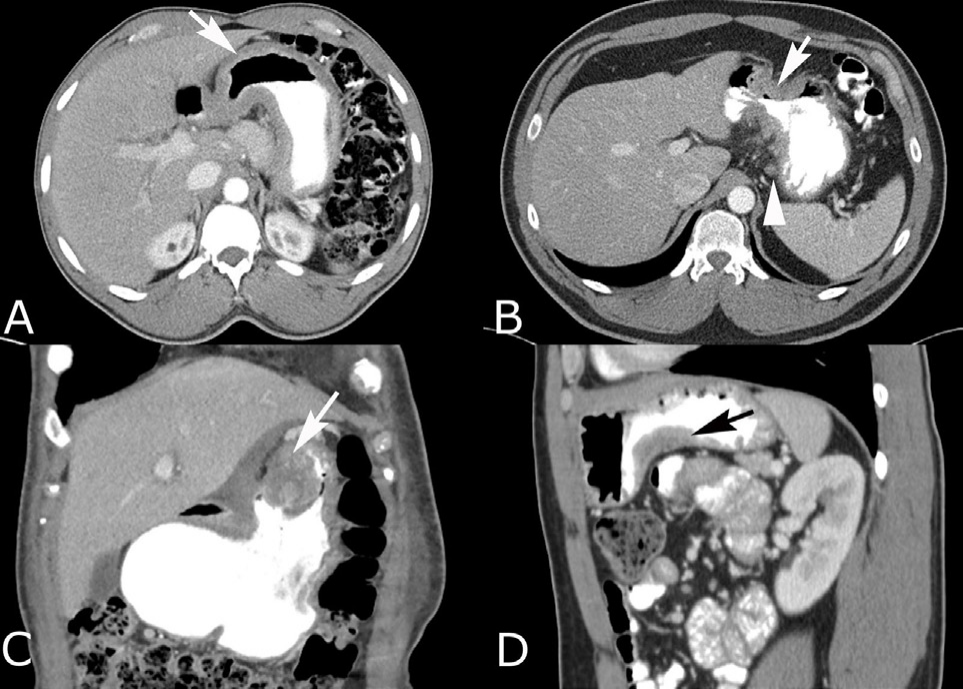
Intravenous and oral contrast-enhanced computed tomography (CT) in four different patients with gastric adenocarcinoma. **(A)** Diffuse gastric wall thickening with loss of normal rugal fold pattern (arrow). **(B)** Focal, circumferential narrowing of the antrum with marked wall thickening that has irregular spiculation into the perigastric fat (arrow). Small perigastric lymph node is present (arrowhead). **(C)** Coronal reconstruction shows an intraluminal polypoid carcinoma (arrow) with heterogeneous enhancement and a soft tissue component that infiltrates the lesser omentum. **(D)** Sagittal reconstruction shows a carcinoma that is producing focal wall thickening (arrow) along the inferior body of the stomach.

**Figure 4:**
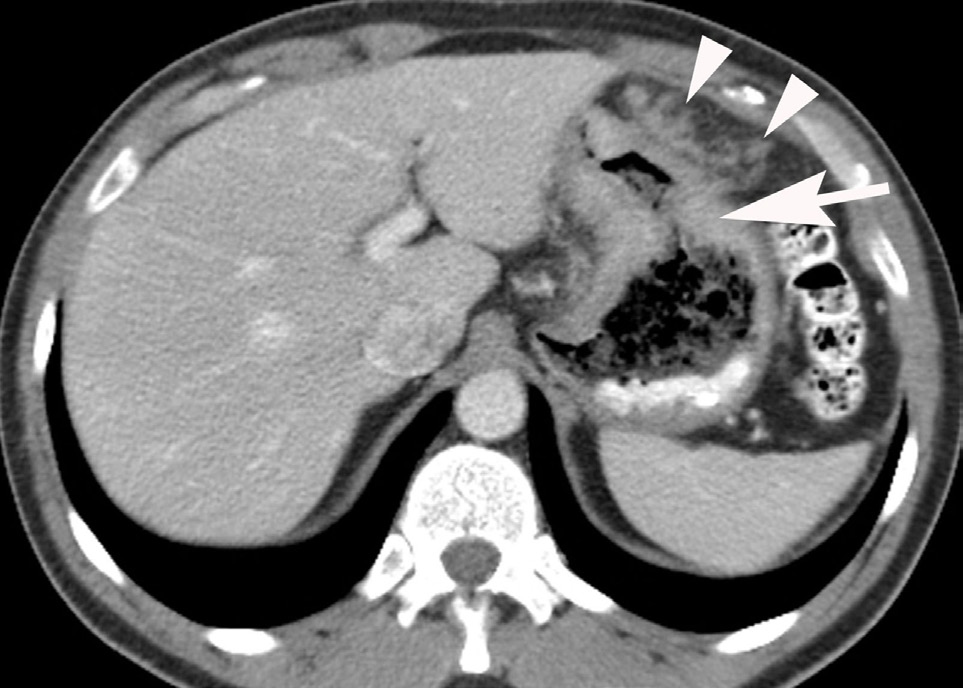
Intravenous and oral contrast material-enhanced computed tomography (CT) showing an adenocarcinoma producing irregular wall thickening of the proximal body of the stomach (arrow) with adjacent omental spread of tumor (arrowheads).

Multidetector computed tomography (CT) is the preferred imaging modality for the diagnosis, staging, and posttreatment surveillance of gastric adenocarcinoma. Its high-resolution, thin slices, and multiplanar capabilities make it an ideal imaging modality to visualize the primary tumor and survey the chest, abdomen, and pelvis for metastatic disease. In patients with suspected or known gastric masses, the stomach should be distended with oral contrast material or water prior to performing CT. Distention of the stomach helps differentiate a collapsed gastric wall from a tumor. The normal gastric wall thickness is usually less than 5 mm in CT and should have a rugal fold pattern. There is no single CT finding that is sensitive or specific for identifying gastric malignancies. It has been shown that the combination of focal or eccentric wall thickening greater than 1 cm, and intravenous contrast enhancement is highly specific (49). The CT appearance of early and advanced gastric carcinomas reflects their macroscopic appearances as defined by the Paris classification for superficial gastric carcinomas and the Borrmann classification for advanced gastric carcinomas. Consequently, early gastric carcinomas appear as polypoid lesions, focal enhancing mucosal thickening, and ulcers. Advanced GC s may manifest as large polypoid masses, varying degrees of wall thickening, and ulcerated and excavated masses (Figure 3) (50). Loss of a normal rugal fold pattern in conjunction with focal or diffuse wall thickening of the stomach are CT features that help distinguish malignant from inflammatory wall thickening (Figures 3 and 4). Coronal and sagittal reconstructions are very helpful to display gastric anatomy and, in some cases, are the most optimal planes for visualizing the tumor. Mucinous tumors may have low attenuation on CT images and may contain calcification.

An irregular, spiculated serosal margin of the tumor is typically seen in gastric adenocarcinoma. This may represent localized desmoplasia or tumor infiltration into the perigastric fat. However, larger, poorly defined linear soft tissue or nodularity extending into or within the surrounding omental and perigastric ligaments is consistent with tumor spread (Figure 4) (51). The findings of adjacent organ invasion include the loss of a normal fat plane between the stomach and the tumor, and extension of the visible tumor into the adjacent organ. In some cases, there is focal enlargement of the adjacent organ. The addition of coronal and sagittal reconstructions improves the prediction of adjacent organ invasion (52).

The accuracy of CT for staging is 66 to 93% (52). The limitations of CT are its inability to detect subtle serosal invasion, metastatic disease in normal-sized lymph nodes, and small peritoneal deposits that may be below the resolution of CT. Positron emission tomography with 2-deoxy-w-(^18^F)fluoro-d-glucose (FDG PET) combined with CT (FDG PET CT) is valuable in some patients for detection of occult disease (53). Accumulation of FDG is typically low in mucinous adenocarcinoma. Combined PET CT has a higher accuracy rate in preoperative staging (68%) than PET alone (47%) or CT alone (53%) (54). Consequently, a combination of diagnostic CT in conjunction with PET or PET CT is essential when PET is obtained.

### Diagnostic Laparoscopy (DL) and Peritoneal Cytology

Diagnostic laparoscopy is strongly recommended as an additional staging tool to overcome some of the previously described limitations of preoperative cross-sectional imaging. In many series, DL was found to upstage 20–25% of patients and can prevent non-therapeutic laparotomies in patients with subradiographic or occult hepatic or peritoneal metastasis (55). The risk of peritoneal disease is much higher in those with linitis plastica and American Joint Committee on Cancer (AJCC) stage T3+, N+ disease; therefore, DL can change the treatment strategy of a patient with suspected stage IV disease. DL can also be utilized to re-assess disease response to systemic therapy, perform peritoneal washings to obtain cytology, or to place a preoperative feeding tube (56, 57).

In addition to performing a DL, analyzing peritoneal cytology for metastatic disease continues to be an area of controversy. Peritoneal cytology that is noted to be positive for tumor cells has been shown to be a poor prognostic marker in the absence of visible tumor spread (C1 disease) (58, 59). Studies have demonstrated that patients with C1 disease have an estimated median survival of only 20 months (57, 58). At our institution, we have a selective approach of using peritoneal cytology in scenarios such as linitis plastica, borderline performance status, or evidence of AJCC T4 disease on imaging.

### Multidisciplinary Treatment Strategy for Operable GC

Patients newly diagnosed with gastric adenocarcinoma should undergo a multidisciplinary stage-dependent treatment strategy that is tailored to the individual patient. This stage-dependent treatment approach should be informed by the following three factors:

(1) The suitability of the patient to undergo curative gastrectomy.
(2) Obtaining accurate pretherapy staging.
(3) The sequence of GC therapy (surgery-first vs. perioperative therapy).

### Suitability to Undergo Gastrectomy

Many variables must be considered prior to undertaking a surgical treatment approach. Evaluation of a patient’s comorbidities and performance status are important preoperative considerations. Managing or minimizing the burden of underlying comorbidities is also important to optimize postgastrectomy recovery and outcomes. While some factors, such as frailty or performance status, may not be overtly changed, other risk factors, including nutritional reserve and electrolyte abnormalities, can be readily corrected. Ongoing investigations suggest that preoperative rehabilitation with outpatient physical therapy prior to surgical treatment may reduce the burden of frailty on operative outcomes (60, 61).

### Obtaining Accurate Pretherapy Staging

An imperative aspect of surgical planning is obtaining appropriate staging information to guide a stage-dependent treatment approach. This can be achieved through preoperative techniques of EUS, contrast-enhanced cross-sectional imaging, and DL as described in the abovementioned sections. When used in combination, these imaging modalities and techniques can be used to determine reliable staging information and allow for the potential of an R0 (margin negative) resection.

### Sequence of GC Therapy

Margin-negative (R0) gastrectomy and adequate lymphadenectomy remain crucial components of operable GC therapy. Because of its additional survival benefit, multimodal therapy (i.e., systemic therapy, with or without radiotherapy) is integrated with surgical therapy. Therefore, most patients with AJCC T2+ operable GC are offered one of the following two treatment sequences: (1) surgery-first, followed by adjuvant chemotherapy and/or chemo radiotherapy or (2) perioperative systemic therapy.

At our institution, we use select patient- and tumor-related factors to inform our decision on the recommended sequence of therapy. Factors that could predict either for residual/recurrent disease after surgery and adjuvant therapy or that could predict for poor patient tolerance or completion of adjuvant therapy after surgery are considered during treatment strategy planning. For example, factors such as proximal GC, presence of linitis plastica, or borderline performance status favor perioperative therapy. Factors that suggest a more aggressive disease that may not be systemically contained well with chemotherapy, such as linitis plastica, similarly favor upfront treatment with chemotherapy as a biologic stress test, before taking the patient to a surgery that will not be beneficial in the long run. In contrast, factors including early stage GC or distal GCs (and their subsequent tumor-related events, including bleeding or obstruction) favor a surgery-first approach.

### Systemic Therapy for Operable GC

The benefit of adjuvant treatment has been established as a means to reduce the recurrence risk of GC following surgical resection. In one meta-analysis, use of any form of chemotherapy as adjuvant therapy to surgical resection of GC demonstrated an 18% overall reduction in the risk of cancer recurrence (62). While no single regimen, treatment sequence, or modality has been established as the adjuvant standard of care, the two approaches are generally considered to be standard options: either a postoperative combination of chemotherapy and radiation therapy or a perioperative use of chemotherapy alone.

#### Adjuvant Chemotherapy and Radiation Therapy

The US Intergroup trial (INT-0116) established the combination of chemotherapy with radiation therapy as one possible adjuvant care standard for operable GC. In this randomized, phase III, open-label trial, patients were eligible for enrollment if their tumor was from stage Ib to stage IVM0 (63). For those patients assigned to the treatment arm, chemotherapy consisted of 5-fluorouracil and leucovorin, administered first as a 5-day cycle alone, then concurrent with radiation on the first four and the final 3 days of treatment, and then for two additional cycles without radiation. In the experimental arm, improvement in overall survival (OS) was statistically significant at 36 months, in comparison to 27 months observed in the control arm (*P* = 0.005).

Several characteristics of patients enrolled in the Intergroup trial bear noting. First, only 64% of patients assigned to the treatment arm of the study were able to complete therapy, highlighting the toxicity of adjuvant therapy, especially in the context of the morbidity and recovery associated with the necessary surgical intervention of gastric adenocarcinoma (64). The compliance rate of adjuvant chemotherapy was higher in patients who underwent subtotal gastrectomy (STG) than in those who underwent TG. Nutritional status or larger body weight loss largely affected the compliance rate. Tumor location and biology could also have been factors in this trial as distal tumor location within the stomach composed 77% of enrolled patients. This trial was also noted for the lack of adequate nodal evaluation in nearly 50% of trial participants, thus raising questions about whether adjuvant therapy compensated for inadequate GC surgery or unrecognized node-positive disease.

#### Perioperative Chemotherapy

Conducted in the UK, the MAGIC trial established an alternative approach to adjuvant therapy for resectable GC through the use of perioperative chemotherapy. This phase III, open-label study randomized patients who were deemed to have a resectable GC in either six cycles of chemotherapy (three each, presurgery and postsurgery) or to observation (65). While patients were eligible if they had a non-metastatic gastric adenocarcinoma of stage II or higher, common practice extrapolates these data to stage Ib tumors as well. Chemotherapy in the MAGIC trial consisted of a triplet regimen of epirubicin, cisplatin, and 5-fluorouracil. Modifications to this regimen, using oxaliplatin instead of cisplatin, and capecitabine instead of 5-fluorouracil, have been shown by the REAL-2 study to be acceptable, affording similar outcomes but with reduced toxicity (66).

In the MAGIC trial, a statistically significant benefit to chemotherapy was established when compared to surgery alone, with 5-year survival rates reported at 36 and 23%, respectively (*P* = 0.009). As is the case with the Intergroup trial, several patient characteristics observed in the MAGIC trial bear noting. First, while 90% of patients assigned to the treatment arm were able to complete the preoperative cycles of chemotherapy, only 57% began the postoperative chemotherapy cycles and only 43% completed them. While again highlighting the challenge of adjuvant therapy administration postoperatively, the relatively consistent ability to administer neoadjuvant therapy is also noted. The majority of tumors were located proximally, including 15% that were at the gastroesophageal junction (GEJ) and 11% that were in the distal esophagus. While the histologic subtypes were not reported in the MAGIC trial either, the more aggressive diffuse subtype tends to favor this proximal tumor location (64). Again, this suggests that the perioperative approach to adjuvant therapy could be optimal for this specific tumor location and histology.

As an alternative perioperative chemotherapy regimen, the combination of 5-fluorouracil, oxaliplatin, and docetaxel has been compared to the combination of epirubicin, cisplatin, and a fluoropyrimidine. Patients in this study had tumors that were clinically staged as above T1 and/or node-positive. Statistically significant overall and progression free survival benefits were observed with the use of the taxane-containing regimen over the anthracycline-containing regimen. This is an alternative chemotherapy combination to consider as perioperative treatment in patients who could tolerate a triplet therapy (67).

#### Additional Adjuvant Approaches

Several other adjuvant approaches to the management of GC have been studied, using the administration of chemotherapy without radiation after surgery. In the Japanese study Adjuvant Chemotherapy Trial of S-1 for Gastric Cancer (ACTS-GC), patients underwent gastrectomy with D2 lymphadenoectomy, followed by either 1 year administration of the fluoropyrimidine S-1 or observation. The 5-year OS rate in the treatment arm was 71.7%, and thus this approach has largely been adopted as standard management in East Asia as a result (68). Noting that, in the control arm, surgery alone resulted in a 61.1% OS has prompted questions as to the generalizability of this approach, since survival rates with surgery alone are typically reported to be much lower. S-1 is not approved for clinical use in the US. Also in east Asia, the capecitabine and oxaliplatin adjuvant study in stomach cancer (CLASSIC) trial compared survival when patients were treated eight cycles of capecitabine and oxaliplatin following surgery with D2 lymphadenectomy versus surgery alone (69). Five-year OS was statistically significant in the treatment arm at 78%, compared to 69% in the control arm (70). As was the case with the MAGIC trial, administration of therapy was difficult, with only 67% of patients receiving all cycles of therapy and 90% of patients requiring some form of dose modification. Again, a higher than expected survival in the control arm has called into question the generalizability of this approach to a non-eastern population, and thus this approach is not widely employed in the western medical world, even though these chemotherapeutics are universally available.

#### Treatment Recommendations

Data from the MAGIC and Intergroup trials cannot be directly compared to each other and thus represent two equally appropriate approaches to adjuvant therapy for resectable GC. In an effort to compare and perhaps combine these two adjuvant approaches, the CRITICS trial has been conducted in Europe (71). In the CRITICS trial, all patients received neoadjuvant chemotherapy (consisting of three cycles of epirubicin, cisplatin or oxaliplatin, and capecitabine) followed by an appropriate surgery. After surgery, patients randomly received either an additional three cycles of the same chemotherapy or concurrent chemotherapy and radiation therapy (45 Gv combined with cisplatin and capecitabine). While the analysis is ongoing, preliminary results presented in 2016 suggest that there is no significant difference in OS between these two postoperative adjuvant approaches (72). Therefore, at least for now, adjuvant management cannot be generalized into one approach, and selection between these standards must consider the individual characteristics of the patient’s tumor.

For patients who have a proximally located, diffuse-histologic subtype of cancer, perioperative chemotherapy akin to the MAGIC approach should generally be recommended. Moreover, given the limitations of reliable administration of postoperative therapy seen in both the MAGIC and Intergroup trials, patients that have an advanced tumor stage (T3/T4 or any node-positive) generally should receive perioperative chemotherapy, so that at least the preoperative cycles of chemotherapy can be administered with some reliability to this cancer that seems to strongly benefit from additional therapy beyond surgery. Conversely, for patients who have a distally located, intestinal-histologic subtype of cancer, postoperative chemotherapy and radiation therapy akin to the Intergroup trial is more appropriate. Additionally, those patients who have a tumor symptomatology that would not allow for surgical delay while neo-adjuvant chemotherapy is administered (such as tumor bleeding or obstruction that cannot be relieved by other methods) should generally proceed directly to surgery. The interplay of these different factors highlights the need for a multidisciplinary consultation to establish treatment sequence and adjuvant plans prior to any therapeutic intervention. While the majority of the time, such multidisciplinary evaluation will conclude that the perioperative approach to therapy is favored, each patient’s unique situation should be individually evaluated. In patients with evidence of advanced disease such as a higher T stage or nodal disease, perioperative chemotherapy should be the recommended adjuvant approach unless there is a clear contraindication.

## Surgical Techniques for Operable GC

### Total Gastrectomy

Total gastrectomy is typically performed in patients with proximal GCs not involving the GEJ (i.e., cancers of the cardia or fundus). The procedure involves removing the entire stomach, GEJ, and omentum, with subsequent intestinal restoration using a Roux-en-Y reconstruction. First, the stomach is carefully dissected and mobilized free of all attachments; then, the pertinent feeding vessels are ligated (at their origin), followed by removal of the stomach. The stomach is transected slightly above the distal esophagus and duodenum, ensuring tumor-free margins, with intraoperative frozen sections confirming normal esophageal and duodenal mucosa (5, 73).

### Subtotal Gastrectomy

A STG is recommended for patients with mid-body or distal GCs and involves resection of approximately 80% of the stomach leaving only a small portion of the proximal stomach. Negative microscopic resection margins, with recommended resection margins greater than 4 cm from the gross tumor, are necessary to ensure an appropriate oncologic resection (43). It is essential to ligate the gastric arteries at their origins, with the exception of the short gastric vessels; these short gastric arteries should be maintained to prevent gastric remnant ischemia. A Roux-en-Y gastrojejunal reconstruction or a loop gastrojejunostomy are options for intestinal continuity. We prefer a Roux-en-Y gastrojejunal reconstruction over a loop gastrojejunostomy to avoid bile reflux to the gastric remnant (5). A hand-sewn or a stapled anastomosis is considered safe and appropriate for the reconstruction.

Level 1 evidence has shown equivalent overall and disease-free survival outcomes after total versus STG for distal GC (overall 5-year survival rate of 62.4 vs. 65.3% for TG vs. STG, respectively) (74). When compared to TG, STG has been shown to provide more favorable nutritional outcomes and quality-of-life measures (75).

### Extent of Lymphadenectomy for GC

Lymphadenectomy with adequate histopathological nodal evaluation are important components of staging and therapy in surgically treated GC patients (76). The extent of lymphadenectomy has been one of the most controversial areas in GC treatment. This controversy has been heavily debated in both Eastern and Western GC therapeutic studies.

There are two types of classifications of lymphadenectomy for operable GC, which are based on: (1) the topographic location of the lymph node stations and (2) the extent of nodal removal, starting within then extending away from the stomach the Japanese Research Society for Gastric Cancer described the topographic classification of histopathological and nodal evaluations. This classification is based on nodal stations within various parts of the stomach and its arterial supply, and extends to the para-aortic nodal region (77, 78). The second classification is based on the extent of nodal removal and is also known using the “D” nomenclature. There are four types of lymphadenectomy: (1) D0 denotes incomplete and is considered an inadequate nodal dissection, unless palliative gastric resection is considered, (2) D1 is defined as resection of perigastric lymph nodes, (3) D2 is referred to as D1+ resection of nodes surrounding the celiac trunk, along with a distal pancreatectomy and splenectomy, and (4) D3 includes D2+ resection of nodes along the hepatoduodenal ligament, posterior surface of the head of the pancreas, and the root of the mesentery (including the superior mesenteric artery and vein) (78, 79).

Two previous large European trials from the UK and Netherlands showed no survival differences between D1 and D2 lymphadenectomy. Both trials also showed worse operative outcomes after D2 lymphadenectomy (80–82). However, long-term results from the Dutch Gastric Cancer Group trial have confirmed a more favorable survival benefit for D2 nodal dissection. Specifically, the 15-year OS rates were 21 and 29%, respectively, for the D1 and D2 group (*P* = 0.34). Lower rates of local (12 vs. 22%) and regional recurrence (13 vs. 19%) were also associated with D2 lymph node dissection (82–84). Although there have been prior studies, contemporary European studies are currently evaluating survival outcomes with D2 compared to D1 lymphadenectomy in the setting of improved D2 operative outcomes (85–87).

To address the controversy around extended lymphadenectomy in Asia, JCOG9501 was a Japanese randomized controlled trial conducted to compare D2 dissection alone versus D2 with para-aortic nodal dissection (PAND) for operable T2b–T4 GC (T2b, T3, or T4). Results from JCOG9501 showed that D2 nodal dissection with PAND does not improve overall and relapse-free survival rates compared to D2 dissection alone (5-year OS rates were 70.3 and 69.2%, respectively) (88, 89). Emerging meta-analyses of D1 versus D2 trials have demonstrated that D2 dissection is associated with a significantly higher postoperative risk, but with equivalent long-term survival rates between D1 versus D2 lymphadenectomy. Additional subanalysis by T stage and spleen/pancreas preservation status detected trends for improved survival with D2 lymphadenectomy in T3/T4 patients and in those with spleen/pancreas preservation (90, 91).

Recently, a robust Cochrane review meta-analysis of over 2,500 patients enrolled in eight Asian or European lymphadenectomy (D1, D2, or D3) GC trials showed no difference in survival between D2 and D3 in Asian lymphadenectomy trials. Furthermore, no significant differences were found in overall and disease-free survival in trials of D1 versus D2 lymphadenectomy. However, D2 lymphadenectomy was associated with a significantly improved disease-specific survival rate compared to D1 lymphadenectomy, albeit with two higher operative mortality rates (92, 93). We speculate that these differences are attributable to differences in disease biology, surgical expertise, variations in where GC surgery is performed (especially in the US), and BMI in Eastern versus Western GC patients (81, 94).

In light of the above, most current Western guidelines recommend at least a D1 lymphadenectomy with a total nodal yield of 15 or more lymph nodes. A modified D2 (also known as pancreas and spleen-preserving D2 lymphadenectomy) is also recommended in expert centers. A D2 lymphadenectomy that can be done safely with a pancreas and spleen preserving technique is also considered the current recommended procedure for both total and STG in select centers (95).

### Minimally Invasive Gastrectomy (MIG) for GC

Minimally invasive gastrectomy has joined the armamentarium of GC surgery. Several non-randomized and observation studies, using propensity score case-matching, have shown that MIG is associated with reductions in surgical site incision pain, length of hospital stay, use of narcotics, and postgastrectomy complication rates (96, 97). The KLASS 01 trial is a Korean prospective randomized trial of laparoscopic versus open gastrectomy for distal GC which has found that laparoscopic distal gastrectomy was associated with decreased morbidity, specifically relating to wound complications (98).

While current evidence suggests that MIG is beneficial for operable GC, several studies have included patients with smaller tumor sizes or early-stage GC. This important criterion should be noted when comparing MIG to open GC surgery, which was likely performed for larger and more advanced GCs.

Currently, there are ongoing large prospective randomized MIG versus open gastrectomy trials in Asia. One large multi-institutional phase III study was initiated in Japan to assess the OS of laparoscopic assisted distal gastrectomy compared to open distal gastrectomy in patients with early-stage GC (99, 100). At this juncture, the efficacy of MIG for GC in the US remains in its infancy and will require additional larger randomized clinical trials to generate additional traction. Furthermore, the dissemination of MIG for GC among surgeons is yet to be fully assessed.

### Endoscopic Resection

While surgical gastrectomy has historically been the gold standard for resection of GC, ER is being used with increasing frequency in the treatment of early gastric cancer (EGC), with multiple reports and societies supporting its implementation (101–108). EGC is defined as GC confined to the mucosa or submucosa irrespective of nodal status (109). The exclusion of nodal status is based on the low likelihood of metastasis among T1a lesions (~0.4% for differentiated, ~4% for undifferentiated); however, there has been pressure to include N0 nodal status, as determined by EUS, in this definition (110). The Japanese Gastric Cancer Association currently recommends ER as an absolute indication for patients with differentiated-type T1a adenocarcinoma of ≤2 cm in diameter and without ulceration (101). More liberal indications for investigational treatment with endoscopic submucosal dissection (ESD) have also been put forth by this organization (see Table 4).

**Table 4:** Indications for endoscopic resection (ER) of early gastric cancer (EGC).

	T1a lesion? (Y/N)	Differentiated type? (Y/N)	Ulceration? (Y/N)	Diameter of lesion (cm)
Absolute indications (ESD or EMR)	Y	Y	N	≤2
**Investigational indications (ESD only)**				
A	Y	Y	N	>2
B	Y	Y	Y	≤3
C	Y	N	N	≤2

*ESD, endoscopic submucosal dissection; EMR, endoscopic mucosal resection*.

*Source: Japanese Gastric Cancer Association, Japanese gastric cancer treatment guidelines, 2010*.

Endoscopic resection can be performed *via* one of the two methods: (1) endoscopic mucosal resection (EMR), where submucosal injection is used to lift the lesion with the surrounding mucosal, which is then removed by snare resection or (2) ESD, where the mucosa of the tissue surrounding the lesion is incised with a high-frequency electric knife and the submucosal layer is then dissected from the muscularis propria under direct visualization. While EMR is an acceptable technique for small lesions (<1.5 cm), ESD is the treatment of choice for most EGC lesions (102). These techniques are complex and require dedicated training to master.

Once ER has been successfully performed, standardized tissue handling and processing protocols, including pinning the specimen and marking borders, are essential to achieve accurate histologic assessment. ER is considered curative when en-bloc resection is achieved with histologic analysis revealing a differentiated-type T1a lesion, negative horizontal and vertical margins, and no evidence of lymphovascular invasion (101). When these standards are not achieved, surgical gastrectomy is recommended. While 10-year follow-up data were recently published showing no difference in all-cause mortality between patients who underwent ER and surgical resection of EGC, there remains concern for a higher rate of recurrence and metachronous cancer in ER patients (111). Despite the need for longer follow-up of these patients to fully understand the impact of ER and further refine inclusion criteria, ER is an organ-sparing, minimally invasive treatment modality that should be offered to appropriately selected patients with EGC.

### Radiation Therapy for Operable GC

Local regional failure following resection for GC is problematic, and preoperative or postoperative radiation therapy reduces the risk of recurrence in selected patients. In a series of patients who underwent a second-look laparotomy following initial curative surgery, 29% of patients had a local regional failure and 88% had a component of local regional failure (112). A more recent study of 1,172 patients who underwent R0 resection for GC identified similar patterns of failure (113). Forty-two percent of patients developed a recurrence and 26% of recurrences were local regional-only recurrences. Local recurrence was associated with a proximal tumor site and male gender.

As previously described, the INT-0116 trial is the largest randomized study to date conducted in the US to evaluate the role of postoperative chemoradiation (63, 114). The updated results of the study demonstrated a strong benefit from chemoradiation. With a median follow-up of over 10 years, chemoradiation was associated with significant improvements in OS and relapse-free survival (HR 1.32, 95% CI, 1.1 to 1.60; *P* < 0.0046 and HR 1.51, 95% CI, 1.25 to 1.83; *P* < 0.001, respectively). Fifty-two percent of patients who received chemoradiation relapsed, as compared to 76% of patients who received surgery alone. There were similar numbers of distance relapses in both arms, which suggests that improved local regional control with adjuvant chemoradiation drove the survival outcomes. Additionally, there was no difference in long-term treatment-related toxicity, including second malignancies, between the two arms. Subset analysis showed a benefit regardless of T-stage, N-stage, location of the primary, or D resection level; however, diffuse-type histology showed minimal treatment effect.

The Korean ARTIST trial tested the role of adjuvant radiation therapy in patients with D2-resected GC (115, 116). The study randomized 458 patients (stage IB–IV) to either six cycles of capecitabine and cisplatin, or two cycles of capecitabine and cisplatin, followed by chemoradiation then two cycles of capecitabine and cisplatin. At a median follow-up of 7 years, there was no significant difference in disease-free survival (HR 0.74, 95% CI, 0.520–1.050; *P* = 0.0922) or OS (HR 1.130, 95% CI, 0.775–1.647; *P* = 0.5272). Local regional failures were reduced from 13 to 7% in the chemoradiation arm. On multivariate analysis, the effect of the addition of radiation therapy on disease-free survival and OS differed by Lauren classification and lymph node ratio. There was also a significant improvement in disease-free survival with the addition of radiation in patients with node-positive disease on subgroup analysis. These findings suggests a benefit of adjuvant chemoradiation in patients with node positive or higher lymph node ratio and intestinal-type GC. The impact of radiation therapy for resectable GC was examined in a large retrospective study from seven US academic institutions (117). Using a propensity score-matched cohort, there was significant OS benefit for patients receiving chemoradiation as compared to chemotherapy alone (46.7 vs. 20.9 months; *P* < 0.001). Patients with N1 disease and those with lymphovascular invasion benefited the most from radiation therapy.

Based on the INT-0116 trial, postoperative chemoradiation remains the standard of care in surgically treated GC patients with T3/T4 or node-positive disease who have not received neoadjuvant therapy. The greatest benefit for adjuvant chemoradiation is with node-positive disease and intestinal-type histology. Building on some of the limitations of the INT-0116 trial, future studies will attempt to better define the role of radiation therapy for GC.

### Multidisciplinary Treatment Strategy for Advanced GC

#### Systemic and Targeted Therapy

In a palliative setting, a number of chemotherapeutic agents have been validated as options for the management of an advanced GC. Noting that this is an incurable condition, consideration of treatment tolerance and quality of life is paramount. Therefore, combinations using two agents rather than three are generally favored for patients who are deemed candidates for systemic therapy. Options for chemotherapy include taxanes, platinum compounds, fluoropyrimidines, the anthracycline epirubicin, and the topoisomerase 1 inhibitor irinotecan (66, 118–121). Beyond conventional chemotherapy, there are few medical therapy options for advanced or metastatic GCs. Available approved biologic therapies are limited to two monoclonal antibodies: the anti-HER2 agent trastuzumab and the antivascular endothelial growth factor agent ramucirumab.

The benefit of trastuzumab was established by the ToGA (trastuzumab for GC) trial (122). In this open-label, phase III, randomized controlled trial, patients were eligible for enrollment if their tumors were found to overexpress the human epidermal growth factor receptor 2 (HER2), which has a role in tumor cell proliferation. When HER2 is expressed by tumor cells, it is reliably bound by the monoclonal antibody trastuzumab, thereby inducing antibody-dependent cellular cytotoxicity and inhibiting HER2-mediated signaling of tumorigenesis. In the ToGA study, all patients were treated with chemotherapy consisting of cisplatin and a fluoropyrimidine; patients were randomly assigned to either additional concurrent therapy with or without trastuzumab. A statistically significant improvement in median OS of 13.8 months was observed with the addition of trastuzumab, compared to 11.1 months in the chemotherapy alone arm (*P* = 0.0046).

Trastuzumab therapy is generally well tolerated. It has the potential to induce reversible cardiac toxicity, however, and thus patient ejection fraction must be monitored while on treatment. There are three major limitations to the value of trastuzumab for treatment of advanced GC. First, the benefit of trastuzumab is achieved when it is paired with chemotherapy, so the patient must endure chemotherapeutic toxicity as well. Second, the additional benefit of trastuzumab, while significant, is slight, with only an additional survival benefit of about 2 months on average. Finally, with only about 10–20% of patients with GCs having a HER2-overexpressing tumor, the extent of application is somewhat limited.

In 2014, ramucirumab was approved for treatment of advanced GC in the second-line setting. This agent achieved two notable firsts: it was the first biologic therapy available to all patients with an advanced GC (without a biomarker limitation), and it was the first anti-angiogenic agent to have single agent utility in any GI cancer (even though it is typically used in combination with chemotherapy). Two studies established the value of ramucirumab in advanced GC. In the REGARD trial, patients whose cancer had progressed following a first-line chemotherapeutic regimen containing at least a platinum or a fluoropyrimidine were randomized to receive either ramucirumab monotherapy or a placebo (123). Patients who received ramucirumab had a statistically significant improvement in median OS of 5.2 months, compared to 3.8 months in the control arm (*P* = 0.047). In the RAINBOW trial, the same enrollment criteria were used (124). Patients were randomized to receive either paclitaxel with a placebo or paclitaxel in combination with ramucirumab. The addition of ramucirumab resulted in a median OS of 9.6 months, which was statistically significant in comparison to the control group, where the median OS was 7.4 months (*P* = 0.017).

Ramucirumab is generally well tolerated, with a panel of side effects that mirrors those already familiar to practitioners from other anti-angiogenic monoclonal antibodies. Commonly, these may include bleeding, thrombosis, hypertension, delayed wound healing, and proteinuria. As with trastuzumab, the benefit of ramuricumab is usually of short duration and, thus, while it can be used as a monotherapy, its benefit is perhaps greatest in combination with paclitaxel chemotherapy.

With no single best sequence for medical therapy administration in advanced GC patient care, selection should consider patient quality-of-life expectations while on therapy, as well as future treatment options. One favored approach would be to use a fluoropyrimidine doublet paired with either oxaliplatin or irinotecan as first-line therapy; if the tumor is HER2 overexpressed, then trastuzumab should be included in the therapy. Such therapy is likely to be well tolerated and beneficial for the majority of patients deemed candidates for systemic therapy. Moreover, when second-line therapy becomes necessary, due to either disease progression or toxicity, this approach will leave the patient naïve to taxane therapy, and thus a good candidate for the paclitaxel/ramucirumab combination. Given the paucity of well-defined treatment sequences and novel agents for GC therapy, enrollment in clinical trials should be encouraged whenever possible.

### Radiation therapy for Advanced GC

Several historical studies have shown the clinical benefits of combined chemoradiation for unresectable or partially resected GC (125, 126). Radiation therapy can also be effective for palliation of symptoms, including pain, bleeding, and dysphagia. A study of 37 patients receiving palliative radiation therapy for GC found that the majority of patients experienced symptom control or improvement following treatment, which persisted throughout their remaining life (127). The combination of palliative radiation with chemotherapy should be considered.

### Palliative GC Surgery

Given that up to 30% of patients present with locally advanced or stage IV GC, surgeons at times are asked to render an opinion about whether or not to offer palliative GC surgery (128, 129). The overarching goals of palliative GC surgery are to improve a patient’s quality of life and allow him or her to continue systemic chemo- or targeted therapy. Patients with advanced disease are typically at a higher risk for malnutrition from possible dysphagia or gastric outlet obstruction. These palliative procedures include gastrectomy or GI bypass, and are often reserved for palliation of ongoing (or pending) bleeding, perforation, or obstruction.

Secondary data analysis from The Dutch Gastric Cancer Trial investigated the impact of palliative STG versus systemic therapy alone on improvement in OS rates (83). Of the 285 patients in The Dutch Gastric Cancer Trial who were not amenable to curative resection, 129 patients did not undergo resection and had either a gastroenterostomy or an exploratory laparotomy alone, while 156 patients underwent a palliative resection. Patients in the palliative resection cohort (>70 years of age with limited metastasis to one other site) had a nearly 3-month survival benefit over patients who underwent a gastroenterostomy or exploratory laparotomy alone at the time of the initial DL for local or metastatic GC. There was, however, a higher morbidity (38 vs. 12%) and longer length of stay (15 vs. 10 days) in the palliative resection cohort, which is likely attributable to the greater extent of surgical resection.

More recent data from Asia has shown that a palliative gastrectomy leads to worse outcomes. In a prospective phase-III trial, gastrectomy followed by chemotherapy did not show any survival benefit compared with chemotherapy alone in advanced GC with a single non-curable factor. Therefore, gastrectomy could not be justified for treatment of patients with these tumors (130). The decision to undergo palliative GC surgery should be informed by the patients’ goals of care, the extent of their disease progression (and non-curable factors), their performance status, and input from the medical and radiation oncology teams.

## Outcomes of Therapy for GC

### OS Outcomes

In the West, most patients diagnosed with GC present with advanced stage disease and carry an overall 5-year survival rate of approximately 20–30% (3). Results from the US National Cancer Database indicate that up to 65% of patients with GC presented with advanced disease (T3/T4), and 85% of these patients fostered nodal metastases at the time of diagnosis (5). Patients who underwent a curative gastrectomy had a median survival rate of 24 months (5-year survival, 20–30%). However, those patients who underwent palliative versus no GC therapy had a median survival rate of 8 versus 5.4 months, respectively (5).

To inform the prognosis of surgically treated GC patients and their clinicians, externally validated patient-centered nomograms are now electronically available to estimate the survival of a patient with GC after a complete (R0) surgical resection. These nomograms are based on the patients’ age, sex, tumor location, tumor size, negative/positive lymph node status, and pathologic features/classification of the tumor(s) (131, 132).

### GC Surgery Operative Outcomes

Mortality rates after TG are noted to be very low (<2%), largely driven by a hospital’s annual surgical volume. A hospital with a higher annual surgical volume (at least 11 gastrectomies per year) was shown to have lower in-hospital mortality by approximately 3–6% when compared with lower volume centers (74, 133). With this understanding and improved surgical techniques, there has been a decline in mortality rates. Declines from 4.5 to 2.3% and from 6.9 to 4.5% have been observed in 30- and 90-day mortality rates, respectively (134, 135).

While operative mortality rates are low after TG, operative morbidity is noticeably higher, with complication rates reported to approach 30–40% (136). These complications can range from systemic (pulmonary embolism, pneumonia, myocardial infarction, deep vein thrombosis) to technically based postsurgical issues (anastomotic leak, anastomotic stricture). If these technical complications do occur, hospital readmission is almost always necessary, requiring drainage of the leaked collection, IV antibiotics, and nutritional support with either total parenteral nutrition or a feeding jejunostomy (134, 137).

Readmission after gastrectomy has been evaluated in many recent studies and estimated readmission rates range from 7 to 20%, with GI complications identified as the most common cause. It was noted that within this subset of patients, those with a higher preoperative nutritional risk and postoperative infections were found to be at the highest risk of complications requiring readmission (34, 137).

### Surveillance

The National Comprehensive Cancer Network recommends that post-GC resection patients should be monitored every 3 to 6 months within the first 2 years of their operation. In addition, it is recommend that their follow-up should include a routine history and physical, upper GI endoscopy (for non-TG patients), cross-sectional CT imaging, and laboratory values, including a CBC, BMP, vitamin B_12_, vitamin D, liver function tests, prealbumin, and iron levels. After 2 years of screening with no signs of recurrence, the routine surveillance intervals may be extended to every 6–12 months. In patients with a genetic predisposition, one should make note of secondary primary malignancies that may arise.

## Conclusion

Despite its declining incidence, GC remains a leading cause of cancer-related deaths worldwide. A multimodal approach to GC is critical to ensure optimal patient outcomes. High-resolution cross-sectional imaging, EUS and staging laparoscopy play important roles in newly diagnosed ostensibly operable GC patients to avoid unnecessary non-therapeutic laparotomies. Margin-negative (R0) gastrectomy and adequate lymphadenectomy remain the backbone of GC treatment that should be performed at high-volume centers. Importantly, adequate GC surgery should be integrated in the setting of a multimodal treatment approach. Treatment for advanced GC continues to expand with the emergence of additional lines of systemic and targeted therapies.

## Author Contributions

HQ contributed to the writing, design, organization, editing, and submission of this manuscript. BS, SM, AP, AM, WC, NH, KU, and AL contributed to the writing and editing of this manuscript. WA-R contributed to the editing, writing, and organization of this manuscript.

## Conflict of Interest Statement

The authors declare that the research was conducted in the absence of any commercial or financial relationships that could be construed as a potential conflict of interest. The reviewer, JP, and handling editor declared their shared affiliation, and the handling editor states that the process nevertheless met the standards of a fair and objective review.
